# Development and evaluation of a novel lateral flow immunoassay for rapid diagnosis of brucellosis across different animal species

**DOI:** 10.1038/s41598-025-08741-5

**Published:** 2025-07-06

**Authors:** Mariam Bedir, Sherif Marouf, Rafik Hamed Sayed, Mohamed El-Diasty, Heba M. Hassan, Momtaz A. Shahien, Samah Eid, Hassan Aboul-Ella, Jakeen Eljakee

**Affiliations:** 1 Animal Health Research Institute (AHRI), Agricultural Research Center (ARC), Giza, 12618 Egypt; 2 Department of Microbiology, Faculty of Veterinary Medicine, Cairo University, Giza, 12211 Egypt; 3 Central Laboratory for Evaluation of Veterinary Biologics (CLEVB), Veterinary Serum and Vaccine Research Institute (VSVRI), Agricultural Research Center (ARC), Giza, 12618 Egypt

**Keywords:** Brucellosis, ASSURED diagnostics, Recombinant protein A, S-LPS-O, Rapid diagnostics, Across species-diagnostics, Sero-diagnostics, LFIA, Microbiology, Applied microbiology, Bacteriology, Infectious-disease diagnostics, Immunology, Applied immunology

## Abstract

Brucellosis, a prevalent zoonotic disease, induces substantial economic losses in livestock due to reproductive impairments and high mortality rates; it also presents considerable public health concerns worldwide. The current work highlights the potential application of the developed lateral flow immunochromatographic assay (LFIA) in the rapid and accurate detection of *Brucella* spp., underscoring its significance in disease management and control. The study investigated the purity of the extracted *B. abortus* smooth lipopolysaccharides-O (S-LPS-O) using high-performance liquid chromatography (HPLC). Moreover, it delved into the development and assessment of a novel diagnostic kit for *Brucella* using a recombinant protein A-lateral flow assay in comparison to the RBPT, SAT, MRT, I-ELISA as screening tests, also to C-ELISA, CFT as confirmatory tests. HPLC analysis indicated a distinctive absence of impurities in the extracted S-LPS-O, contrasting with the three peaks observed in the commercial LPS chromatogram. Sensitivity, specificity, and accuracy evaluations were performed, with the LFIA demonstrating promising results, showcasing its potential as a reliable diagnostic tool. Statistical analysis of the LFIA, RBPT, SAT, MRT, ELISA, CFT, and PCR results revealed notable sensitivity, specificity, and accuracy rates, affirming the diagnostic efficacy of the LFIA. The novel layout of the LFIA and the purity of extracted S-LPS-O showcased significant progress in the diagnostic capabilities of the developed LFIA. Moreover, the currently developed LFIA introduces a rapid and reliable diagnostic tool, particularly in resource-limited settings, owing to its ease of use and minimal equipment requirements with its competitively obtained results.

**Supplementary Information:**

The online version contains supplementary materialavailable at 10.1038/s41598-025-08741-5.

## Introduction

More than a century has passed since David Bruce discovered the agent that causes Malta fever, and brucellosis is still one of the major zoonotic illnesses that cause significant cattle reproductive failure and financial losses in the dairy industry with global distribution^1^. A classic example of a zoonotic disease that is common worldwide and has been identified by the World Health Organization (WHO) as the most neglected zoonotic disease, brucellosis, which is caused by several species of the genus *Brucella*^2^. It is still a major epidemic in low- and middle-income countries^3,4^. It is more frequently associated with the employment and intake of animal-based meals^5^. Consequently, the largest risk groups include veterinary professionals, animal farmers, and slaughterhouse workers, who typically contract infections through mucous or abraded skin^6^. People who breathe in contaminated dust or airborne droplets, such as those who work in the fur processing industry, are susceptible to respiratory tract diseases. Most often, contaminated food or water causes gastrointestinal tract infections in people who have never been around farm animals or animal products^7^.

In terms of endemic regions and time, the burden of brucellosis infections varies. Despite this dynamic, the one constant in the chain of transmission is that human cases of brucellosis are invariably associated with certain animal reservoirs. The course and outcome of brucellosis are greatly impacted by early detection and laboratory confirmation of the diagnosis because the disease typically manifests in a variety of non-specific ways. Since brucellosis cannot be identified without solid microbiological, molecular, and epidemiological data, a coordinated approach to diagnosis and control is always necessary. Controlling brucellosis in animals and, by extension, humans, requires an accurate diagnosis. One of the main challenges to eliminating brucellosis is accurate diagnosis^8^. Because of the significant risk of laboratory-acquired infections, routine identification and discrimination of brucellosis-suspected specimens based on culture isolation and phenotypic characterization necessitates biosafety level 3 (BSL-3) techniques^9^.

Brucellosis management presents several diagnostic difficulties^10^. First, the infection may spread and become sustainable if it spreads to a larger variety of animal hosts through culture-based spillover^11^. The management of this persistent and widespread infection necessitates the creation of diagnostic tests that can get past the drawbacks of current methods, such as the requirement for sophisticated equipment and skilled technicians, which are unavailable in many diagnostic situations^12^, and low sensitivity and/or specificity, which are additional shortcomings that could produce unreliable results. Diagnostic methods for brucellosis still commonly include SAT, RBPT, CFT, and ELISA^13^. For more accurate identification and to prevent false positives, it is often advised to utilize the RBPT in conjunction with other common serological tests. Although CFT was created to detect IgG, its cross-reactivity with calves who have received the *B. abortus* S19 vaccine makes it primarily a confirming test^14^. Because ELISA has better sensitivity and specificity than SAT, it is frequently used to identify partial antibodies and diagnose chronic cases of brucellosis^15^.

To enhance diagnostic capabilities, a variety of PCR-based assays have been created for the identification of *Brucella*. When taken as a whole, the test repertoire covers several diagnostic process facets. Differential PCR-based tests are typically more complicated and, as a result, more challenging to execute. Numerous methods, such as locus-specific multiplexing (AMOS-PCR based on IS711), PCR-RFLP (the omp2 locus), arbitrary-primed PCR, and ERIC-PCR, have been investigated to distinguish between *Brucella* species and strains^16^. A verification test is frequently necessary, and none of the diagnostic methods that have been developed and are currently accessible satisfy the conventional criteria for a convincing diagnosis. Additionally, none of the assays are advised to be used alone in endemic areas^17–19^. The LFIA, which is used to identify immunoglobulin IgM and IgG-specific antibodies (Ab) against LPS, is one of the quick and accurate diagnostic tests^20–23^.

This study’s objective is to:

1. Develop and evaluate a novel sero-diagnostic with the affordability, sensitivity, specificity, user-friendliness, rapid and reliable performance, equipment-free operation, and deliverability to those in need (ASSURED) criteria for brucellosis diagnosis in comparison to PCR and a wide range of conventional serological assays (RBPT, SAT, MRT, and I-ELISA as screening tests and C-ELISA and CFT as confirmatory tests).
2. Illustrate how the purity of LPS and non-specific immunoglobulins significantly impact the diagnostic specificity and sensitivity of sero-diagnostics and offer a promising way to mitigate this impact using the newly designed assay’s suggested format.
3. Improve the common sero-diagnostic LFIA based on the conjugates of immunoglobulin-binding proteins with traditional nano-dispersed labels that ensure an increase in labeled immune complexes in the test line which by its role reflects on the newly developed LFIA sensitivity and range of detection to a different and wide range of *Brucella* spp. in different animal species by the same developed diagnostic assay.

## Materials and methods

### Study design and laboratory animal’s source and fate

All laboratory animals involved in the current study were purchased from a commercial private laboratory animal farm in El-Fayoum governorate, Egypt, and hosted by the laboratory animal unit at the Animal Health Research Institute (AHRI), Agriculture Research Center (ARC). Laboratory animals that died during the study were bio-safely disposed and the rest of the laboratory animals that remained alive after the end of the experiment were neither released nor euthanized, they are kept, and properly managed till being reused, reassigned in other experiments, or naturally died, and then bio-safely disposed of.

### Pre-kit-development stage (preliminary stage)

#### Brucella strain

Reference *B. abortus* strain S99 (vaccinal strain) was obtained from the Animal Health Research Institute (AHRI), Agricultural Research Center (ARC), Egypt.

#### Extraction of smooth lipopolysaccharides – O (S-LPS-O)

Reference *B. abortus* strain S99 (vaccinal strain) was cultured on *Brucella* agar medium in 40 plates and incubated for 48 h at 37 °C. Next, 5 ml of *Brucella* broth was taken in a sterile syringe without the needle and used for washing the bacteria from the plates’ surface, followed by collection of the bacteria in the liquid broth to obtain the bacterial suspension which was lysed by heating for 30 min in a water bath at 80 °C after checking the broth’s purity and homogeneity^24,25^. Before the extraction process, proteinase K, DNase, and RNase treatments were carried out with minor adjustments to get rid of contaminating protein and nucleic acids. Proteinase K (100 µg/ml) (ROCHE, MANNHEIM, Germany) was added to the cell mixture for this purpose, and the tubes were then maintained at 65 °C for an extra hour. Following this, the mixture was treated with 40 µg/ml of RNase (ROCHE, MANNHEIM, Germany) and 20 µg/ml of DNase (ROCHE, MANNHEIM, Germany) in thepresence of 1 µl/ml of 20% MgSO_4_ and 4 µl/ml of chloroform. The mixture was then incubated for the entire night at 37 °C. Centrifugation at 12,000×g for 30 min at 4 °C was performed to guarantee the bacterial death. The bacterial sediments were stored in sterile tubes^26,27^.

Then, 30, 50, 75, and 100 mg (255 mg) of wet bacterial sediment were dissolved gradually and completely in 510 µl distilled water achieving a 1:2 wet bacterial sediment: distilled water ratio, followed by boiling the resulting suspension to 68 °C. Next, 570 µl of warmed 90% phenol was added to the suspension and agitated for 30 min at the same temperature achieving a 20:45 wet bacterial sediment: warmed 90% phenol ratio^22,26^. The suspension was centrifuged at 4 °C for 15 min at 8000×g after its temperature quickly lowered to 5 °C in an ice bath. The four phases that followed were then obtained: Phenol-saturated aqueous was present in the top phase, followed by white sediment in the second layer between the top and aqua phases, aqua-saturated phenol in the third phase, and sediment in the tube’s lower portion in the final phase. After being thoroughly separated, the first aqueous and second sediment phases were eliminated. The phenol phases were then carefully separated, and a half volume of cold methanol was added to it for the deposition of the proteins and nucleic acids (unlike other bacteria, LPS in *Brucella* enters the phenol phase)^22,24,25^.

The samples were then kept at 4 °C for 30 min, and after that, they were centrifuged at 4 °C at 1500×g for 10 min. The top phenol phase was then separated, the protein and nucleic acid sediments were disposed of 50 mg/ml of HCL was added, and the mixture was stirred for 15 min at 56 °C. After centrifuging the mixture for 10 min at 4 °C at 1500×g, the supernatant was gathered, and the leftover protein sediment was disposed of. Three volumes of methanol (99 volumes of methanol plus 1 volume of sodium acetate-saturated methanol) were added, and the mixture was agitated for 1 h in an ice bath to produce the S-LPS-O. The S-LPS-O was collected and deposited by centrifugation at 8000×g for 20 min at 4 °C. The precipitated S-LPS-O was dissolved in 25 µl of distilled water, to which 75 µl of cold methanol reagent was added. The mixture was vortexed for 60 min at 27 °C and then centrifuged at 8000×g for 20 min at 4 °C to remove any leftover phenol. A spectrophotometer was then used to measure the silt content after the pellet had been dissolved in 500 µl of distilled water^26^.

#### Verify the proper extraction of the prepared S-LPS-O

##### High-performance liquid chromatography (HPLC)

The proper extraction confirmation of the prepared S-LPS-O was determined by using HPLC^27^. Standard preparation of LPS of *Salmonella* Typhimurium was used as standard LPS and the chromatographic separation of both the standard LPS and the extracted S-LPS-O was compared^28^.

##### Rabbit pyrogenic assay

Two white New Zealand rabbits weighing between 1.7 and 2.3 kg were used. One rabbit was given an intravenous injection of 5 ng/kg of body weight of the purified S-LPS-O^27^, and the second rabbit was injected with sterile PBS as a placebo control. Temperatures were measured with indwelling rectal thermostats and recorded before injection and 4 h after pyrogen administration^29,30^.

#### Preparation of emulsions used for animal immunization

The priming immunization emulsion was made by mixing equal amounts of the previously prepared S-LPS-O and complete Freund’s adjuvant (CFA, SIGMA-ALDRICH, USA) using two syringes and a connector for 20 min until the mixture reached a milky white, viscous, creamy emulsion that was stable after overnight testing at 6 °C in the refrigerator. This was the procedure used to prepare the priming immunization emulsion for S-LPS-O antigen. After overnight testing at refrigerator temperature (6 °C), the boostering immunization emulsion was made up of equal volumes of incomplete Freund’s adjuvant (IFA, SIGMA-ALDRICH, USA) and the previously prepared S-LPS-O. It was thoroughly mixed using two syringes and a connector for 20 min until it reached a milky, white, viscous, and creamy emulsion with a stable formulation. Complete aseptic conditions were used to produce and administer both kinds of vaccination emulsions^31^.

#### Laboratory animal selection and immunization protocol design

##### The selected laboratory animal

Two white male New Zealand rabbits, each weighing two kilograms, were intended to serve as the bio-factory for the synthesis of S-LPS-O-polyclonal antibodies (S-LPS-O-pAbs). A fully developed animal will guarantee a fully functional immune system, which is essential for the manufacturing of antibodies. Additionally, because of their small size, high affinity, relatively long lifetime, ease of getting blood, robust immune response, and low cost of housing, rabbits were selected as the laboratory animal from the list of regularly used lab animals utilized for antibody manufacturing^32^. To increase the amount of blood samples that contained antibodies for each antigen (Ag) and to account for the possibility of one of the two animals unexpectedly dying before the injection procedure was finished, the identical S-LPS-O Ag was injected into two animals.

##### The design of the animal immunization protocol

The animal vaccination protocol was designed and executed based on^33^, along with routine monitoring of the production of antibodies utilizing an agar gel precipitation test (Ouchterlony’s test) Table 1.

**Table 1:** Illustration of a detailed animal immunization schedule.

Two-weeks acclimation period	
Day Zero	A blood sample was collected from each animal involved in the conducted study
**Day 1**	**0.5 ml/kg of the priming emulsion**,** the whole dose is injected intradermally (I/D) in different sites**
Pre-injection	Ear vein blood sampling and agar gel testing as a follow-up for Abs production
**Day 14**	**0.15 ml/kg of the boostering emulsion**,** the whole dose is injected subcutaneously (S/C) in different sites**
Pre-injection	Ear vein blood sampling and agar gel testing as a follow-up for Abs production
**Day 28**	**0.15 ml/kg of the boostering emulsion**,** the whole dose is injected subcutaneously (S/C) in different sites**
Pre-injection	Ear vein blood sampling and agar gel testing as a follow-up for Abs production
**Day 32**	**0.15 ml/kg of the boostering emulsion**,** the whole dose is injected subcutaneously (S/C) in different sites**
Pre-injection	Ear vein blood sampling and agar gel testing as a follow-up for Abs production
**Day 46**	**0.15 ml/kg of the boostering emulsion**,** the whole dose is injected subcutaneously (S/C) in different sites**
Pre-collection	Ear vein blood sampling and agar gel testing as a follow-up for Abs production
**Day 56**	**Final blood collection**

#### Separation and purification of the obtained pAbs

Blood samples were obtained separately and stored at room temperature (25 °C) in a slightly oblique position for one hour without disturbance. They were then refrigerated for an additional hour at 6 °C. Following a 30-minute 10.000×g centrifugation of 25 ml of blood at room temperature which was performed as follows; in three consequence stages 1st centrifuging the collected blood for 5 min at 10.000×g, then discarding the pellet, centrifuging the obtained supernatant for 10 min at 10.000×g, discarding the pellet, and finally, centrifuging the obtained supernatant for 15 min at 10.000×g, then discarding the pellet. This preparatory stage is very important as it helped in obtaining better Abs extraction by the meaning of purity and concentration. 2.02 ml of caprylic acid was added gradually to 25 ml of rabbit serum while being stirred for 30 min at 25 °C. After centrifuging the mixture for 20 min at 10.000×g, the pellets were discarded, and the supernatants were gathered. Using 12.000–14.000 molecular weight cut-off (MWCO) dialysis bags (SIGMA-ALDRICH, USA) with three buffer changes, the collected supernatants were dialyzed separately against phosphate-buffered saline (PBS) at 4 °C overnight. The concentration of the resulting immunoglobulins was then determined. The concentrations of immunoglobulin that were collected varied between 1.1 and 1.4 g/dl. After that, an ultrapure water dilution was used to achieve a concentration of 1 mg/ml^34^.

#### Agar gel precipitation test (Ouchterlony’s test) optimization for evaluating the separated pAbs

Agar gel plates were made by dissolving 0.9 g of agarose in 100 ml of PBS, heating the mixture for two minutes, and then pouring 5 ml of the dissolved agarose into 5 cm plates. This will lead to a 5 mm agar gel thickness and a 0.9% concentration. The plates were then allowed to solidify. Throughout the current work, the agar gel test was employed in many contexts: first, to track antibody production throughout the planned immunization regimen; second, to assess the impact of the pAbs purification procedures^31^.

### Kit-development stage (development and standardization stage)

#### Preparation of colloidal gold (CG) nanoparticles (NPs)

After 50 ml of ultrapure water was brought to the boiling temperature (100 °C) on a hot plate, 0.5 ml of 0.2% HauCl_4_·3H_2_O (Gold III chloride trihydrate, SIGMA-ALDRICH, USA) was added after rapid magnetic stirring. One ml of 1% (w/v) sodium citrated buffer was rapidly added while the mixture was boiling vigorously. The combination started as a colorless solution that turned black and then ruby red after about two minutes. It was then stirred continuously and allowed to boil at 100 °C for an additional 10 min^31,34,35^. A UV-Vis spectrophotometer and transmissible electron microscope (TEM) imaging were used to measure the diameter of the produced nanoparticles in a range of 400–600 nm.

#### Conjugation of the Recombinant protein A with the colloidal gold nanoparticles (Au NPs-recombinant protein A coupled bio-conjugate)

The colloid gold solution was adjusted to pH 8.5 with 0.02M K_2_CO_3_. With gentle stirring, 100 µl of the recombinant protein A (BIOSCIENCE, Sweden) (1 mg/0.1 ml of 0.05% NaCl buffer) was added dropwise to 10 ml of pH-adjusted colloid gold solution. The mixture was gently mixed for 10 min, blocked with 1% (m/v) final concentration of polyethylene glycol (PEG − 20,000 kDa) followed by stirring for an additional 15 min and centrifugation at 10,000×g for 30 min. The pellets were suspended in 1 ml dilution buffer [20mM Tris/HCI buffer (pH 8.2) containing 1% (w/v) BSA, 3% (w/v) sucrose, 0.02% sodium azide and stored at 4 °C until used^36^.

#### The segmental and micro structuring of the newly developed kit

##### Preparation of the lateral flow solid phase (multi-laminated membrane strip)

**Fig. 1:**
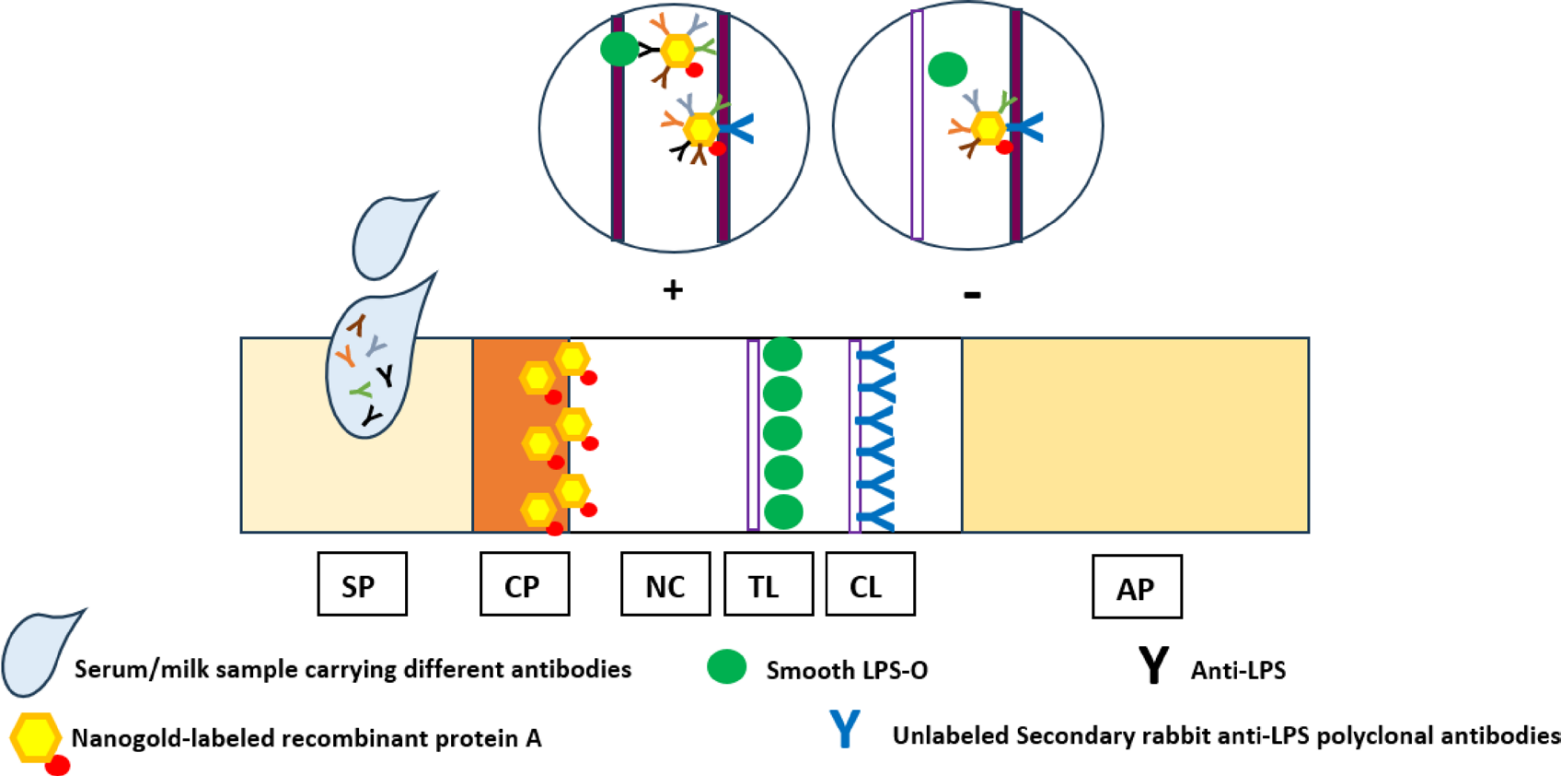
Illustrative diagram of the positions of each incorporated bio-reactant in the structure of the newly developed kit as well as a focus on the bio-reactant layout in both positive and negative results. *AP* absorbent pad, *CL* control line, *TL* test line, *CP* conjugate pad, *SP* sample pad, *NC* nitrocellulose membrane.

To prepare the newly designed LFIA solid phase, the sample pad (SP, AHLSTROM, UK) was composed of glass fiber, soaked with a pH 7.2 PBS solution that contained 0.3% Tween-20 and 0.5% (w/v) triton X100, and then dried at 37 ºC. After that, it was stored at room temperature in a dry environment until it was needed. Conjugate pad (CP, AHLSTROM, UK): composed of glass fiber, it was dried at 60 °C after being treated for 10 min with 0.1% Tween-20. After cutting the produced glass fiber into 3 cm × 0.5 cm sections, 150 µl of colloidal gold probe (recombinant protein A coupled with nanogold) was added. The conjugate pad was kept dry at 4 °C after being dried for an hour at 37 °C. Nitrocellulose membrane (NC, BIODOT-XYZ-3, USA): Ag–Ab immune complexes are generated and observed on microporous nitrocellulose membranes, which serve as the carrying matrix. The purpose of the cotton fiber pad roll known as an absorbent (wick) pad (AP, AHLSTROM, UK) is to collect the liquid that is processed through the strip by capillary action^34^. Adhesive Polyvinyl Chloride (PVC, AHLSTROM, UK): A four-row laminated adhesive membrane that serves as the carrier base for the remaining lateral flow components. The process begins with removing the sticker from the second row and attaching the NC, then moving on to the first row and attaching the AP, then moving on to the third row and attaching the CP, and lastly removing the sticker from the fourth row and attaching the SP^31^ Fig. 1.

##### Loading of the different bio-reactants on the prepared lateral flow solid phase

**Fig. 2:**
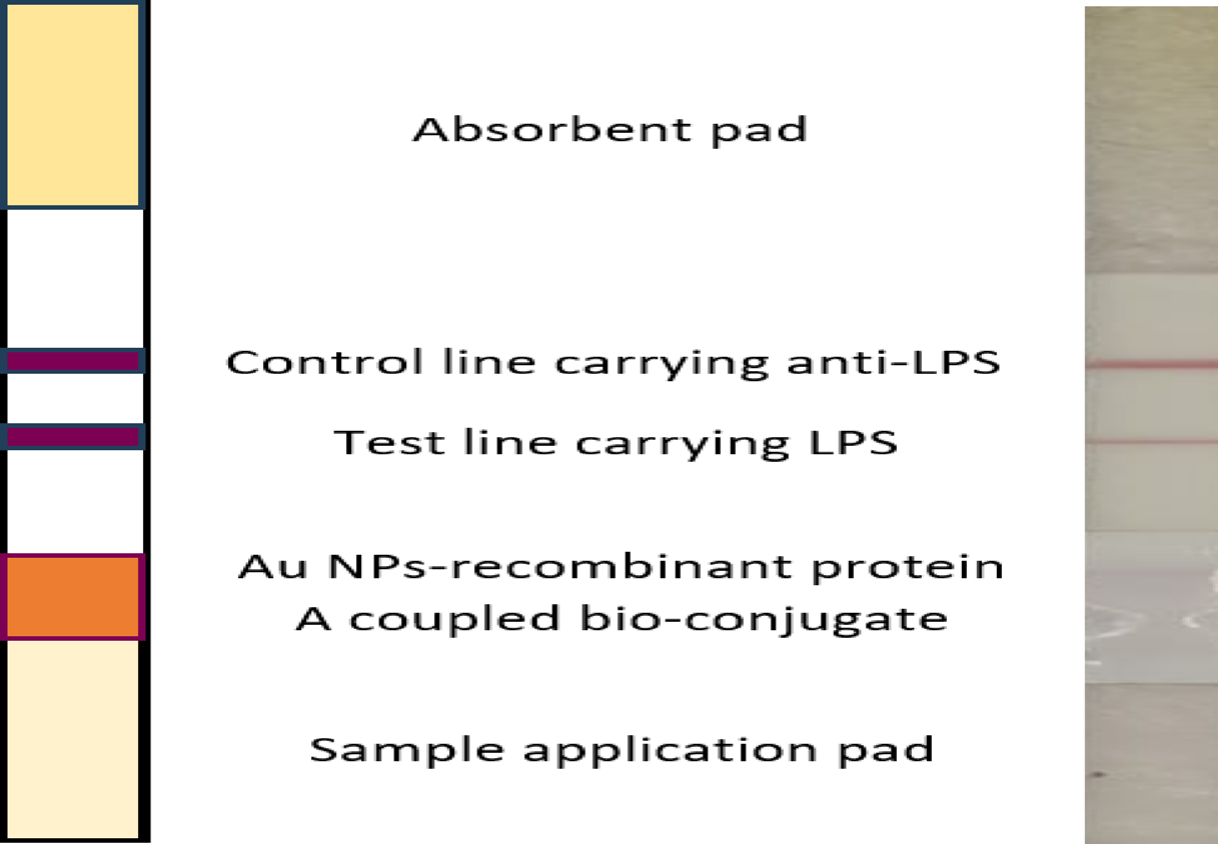
Coupled diagrammatic and real structure of the developed kit.

Coating S-LPS-O and rabbit anti-S-LPS-O IgG pAbs were optimized to yield clear and accurate results. The ruby red on the C line was visible when the concentration of rabbit anti-S-LPS-O IgG was more than 0.3 mg/ml and became clearest as the coating concentration increased to 0.5 mg/ml. As for the T line, the color line and uniform distribution were observed when the coating concentration was more than 1 mg/ml. To meet the requirements of the test strip observation, 1.5 mg/0.1 ml of S-LPS-O and 0.5 mg/ml of the polyclonal rabbit anti-S-LPS-O IgG antibody (pH 7.4) were chosen as the optimal concentration. The antibody printer (ISOFLOW, USA) was used to load the 1 µl/ 1 cm line of unlabeled rabbit anti-S-LPS-O antibodies on the control zone. In comparison, the test zone was loaded with a 1 µl/ 1 cm line of S-LPS-O^35^ Fig. 2.

#### Cutting of the test strips and kit assembly in lateral flow cartilage

The loadedadhered to the PVC card developed lateral flow sheets, automatically were cut by an automatic Zeta Programmable Guillotine Cutter GCI-800-Lab (ZETA corporation, UK) achieving 0.5 cm width per strip, then due to the research level production quantity, manual assembly, and packaging of the strips into the lateral flow cartilage has occurred. The used cartilage is characterized by (S) letter labeled sample application window and (T and C) letter labeled window marking the test and control spots on the strip^31,34,35,37^.

#### The newly developed kit procedures and limit of detections (LoDs) determination

**Fig. 3:**
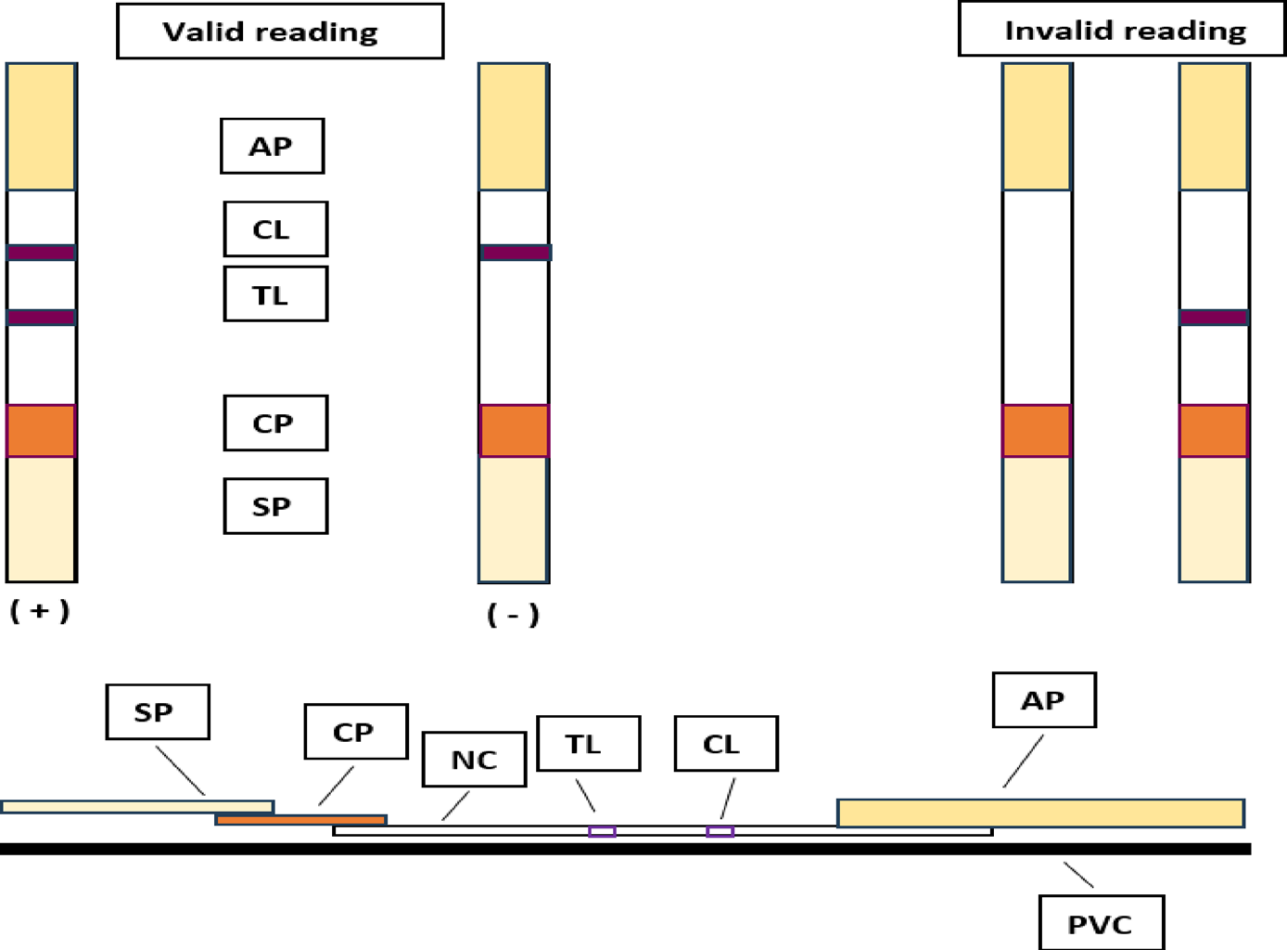
Illustrative diagram of the layout of the newly developed kit and illustrations of valid and invalid readings. *AP* absorbent pad, *CL* control line, *TL* test line, *CP* conjugate pad, *SP* sample pad, *NC* nitrocellulose membrane, *PVC* polyvinyl chloride card.

Two milk/serum drops were placed on the sample pad through the sample application window, then the kit was left on a flat horizontal surface and observed for 5 min. Two ruby red lines on both the test and control zones indicate a positive sample while only one ruby red line on the control zone indicates a negative sample. If the tested samples contained a lower Ab concentration against *Brucella* than the detection limit, only one band could be visualized in the control zone. Otherwise, if no band was seen at the control and test zones, it indicated the invalidity of the test. The concentration of Ab against *Brucella* or LPS in the tested samples controlled the intensity of the test line in direct proportion. The control zone served as positive control and the sign of validity to confirm the migration of functional, conjugated antigens in the system Fig. 3. The total time required for the test was < 5 min. The estimation of the results of the test strip could be performed visually with the naked eye. The LoDs were determined by testing the developed kit by serially *B. abortus* and *B. melitensis* standard antisera.

### Post-kit-development stage (evaluation stage)

#### Collection of the samples used during the newly developed kit field challenge

A total of 950 samples were collected and utilized during the newly developed kit evaluation stage as follows: 870 serum samples (460 bovine samples, 400 ovine samples, and 10 swine samples) and 80 milk samples (40 bovine samples and 40 ovine samples) were collected randomly from various farms. These samples were sourced from diverse farms across Mansoura, Sharkia, and Giza governorates, Egypt. The samples were incorporated into this study after informed consent was obtained from the farms’ manager and owner. All laboratory analyses were conducted at the Animal Health Research Institute (AHRI) and the Central Laboratory for Evaluation of Veterinary Biologics (CLEVB), Veterinary Serum and Vaccine Research Institute (VSVRI).

#### Rose Bengal plate test (RBPT)

All collected serum samples were tested using the RBPT as follows; the test reagents and samples were brought to room temperature (18–25 ºC). Resuspend the antigen vial (Bacterial suspension of *B. abortus* stained with Rose Bengal and buffered at pH 3.6. containing 0.95 g/l of sodium azide) gently. Aspirate the dropper several times to obtain a thorough mixing. Place 1 drop (50 µl) of the serum under test into one of the circles on the RBPT card. Dispense 1 drop of positive control serum (Anti-*Brucella* animal serum with an agglutinating activity of approximately 100 IU/ml contains 0.95 g/l of sodium azide) and 1 drop of negative control serum (Animal serum with an agglutinating activity < 10 IU/ml contains 0.95 g/l of sodium azide) into two additional circles. Add 1 drop of Rose Bengal Ag to each circle next to the sample to be tested. Mix the contents of each circle with a disposable stirrer while spreading over the entire area enclosed by the ring. Use separate stirrers for each mixture. The card rotated slowly by hand for 4 min. The results were observed immediately under a suitable light source for any degree of agglutination. Nonreactive was described as a smooth suspension with no visible agglutination, as shown by negative control. Reactive was described as any degree of agglutination visible macroscopically^38–42^.

#### Serum agglutination test (SAT)

The following procedure was used to test all obtained serum samples using the SAT: 0.9 ml of saline containing 0.5% phenol was pipetted into the first test tube, and 0.5 ml of the same solution was pipetted into the remaining test tubes. 0.1 ml of checking positive serum was put into the first test tube in the first row, and 0.1 ml of the tested serum was added to the first test tube in the second row. After carefully mixing the serums in the first test tubes, 0.5 ml from each test tube was transferred to the second one in the row. The final test tubes underwent this process one more, and the 0.5 ml of diluted serum surplus was disposed of. In this manner, the first test tube’s serum dilution is 1:10, the second’s is 1:20, and so forth. The serum dilutions made in this manner were then supplemented with 0.5 ml of the antigen diluted with 5% NaCl solution in the working dilution of 1:9. Following a good shake, the test tubes were incubated for 24 h at 37 °C. They were then allowed to stand for 60 min before the response was measured. Evaluation of the obtained results and test interpretation was performed as follows; positive: agglutination, complete clarification or only slight opalescence of the supernatant, and negative: no agglutination, milky turbidity^43^.

#### Complement fixation test (CFT)

All collected serum samples were tested using the CFT as follows: Veronal buffer preparation for CFT, guinea pig complement, hemolytic serum, negative control, and positive control, respectively were performed in the Animal Health Research Institute. The CFT was carried out in accordance with the OIE method. Test sera that were not diluted were inactivated for 30 min at 60 °C in a water bath. 25 µl of inactivated and 1/5 diluted test sera were added to each row of 96-well microtiter plates. The wells in rows B, D, F, and H received 25 µl of antigen diluted to working strength (1/10), while the anti-complementary rows (A, C, E, and G) received an equivalent volume of veronal buffer. All control wells and the antigen-test sera mixture were incubated for 30 min at 37 °C. After that, each well received 25 µl of the working complement of the tested strength, which was then incubated for 30 min at 37 °C. Separate plates were used to handle each control properly. Next, a 25 µl hemolytic serum diluted 1:1000 in 1% sheep red blood cell (SRBC) was also added to each well. For 30 min, the plates were incubated again at 37 °C. Before reading the data, the plates were left at 4 °C for two to three hours to give the unlysed cells time to settle. Anti-complementarity wells were used to compare the percentage of lysis. Positive outcomes are defined as RBCs settling of 50% or more, whereas negative results are defined as lyses of 50% or more^41,42,44^.

#### Indirect enzyme-linked immunosorbent assay (I-ELISA)

All collected serum samples were tested using the I-ELISA as follows: briefly, all sera samples, controls, and reagents were brought to room temperature (18–25 ºC). To test sera samples, controls were incubated with *B. abortus* LPS-coated plates at room temperature for 45 min. After cleaning, each well in the microplate received 100 µl of multispecies Horseradish Peroxidase (HRP) conjugate (anti-species Abs-HRP labeled Abs), which was then allowed to sit at room temperature for half an hour. A Tetra methyl Benzidine (TMB) substrate solution was administered and left in a dark area for 15 min after being cleaned to get rid of any extra conjugate. The amount of specific antibodies in the sample to be examined was directly correlated with the color that resulted. Lastly, an ELISA microplate reader (Lab system, USA) operating at 450 nm was used to read the plates. The formula suggested by the kit manufacturer (IDEXX Brucellosis Serum Ab Test, USA) was used todetermine the percent value (S/P%)^41,42,45^.

#### Competitive enzyme-linked immunosorbent assay (C-ELISA)

All collected serum samples were tested using the C-ELISA as follows: briefly, all sera samples, controls, and reagents were brought to room temperature (18–25 ºC). Sera samples were to be tested, and controls were incubated at room temperature for 45 min with *B. abortus* LPS-coated plates. Following washing, 100 µl multispecies HRP conjugate (anti-LPS-HRP labeled Abs) was dispensed to each microplate well and kept at room temperature for 30 min. After washing to remove excess conjugate, a TMB substrate solution was dispensed and kept in a dark place for 15 min. The resulting color developed inversely proportioned to the amount of specific antibodies present in the sample to be tested. Finally, plates were read using an ELISA microplate reader with a 450 nm wavelength (NE LABSYSTEMS, USA). The percent value (S/P%) was calculated using the formula recommended by the kit manufacturer (IDEXX Brucellosis Serum Ab Test, USA) kit protocol^46,47^.

#### Milk ring test (MRT)

All collected milk samples were tested using the MRT as follows: the milk samples and antigen were brought to room temperature (18–25 °C). The antigen bottle was gently shaken well. The test was performed by adding 30 µl of antigen to a 1.5 ml volume of whole milk. The height of the milk column in the tube must be at least 25 mm. The milk samples must not have been frozen, heated, subjected to violent shaking, or stored for more than 72 h. The milk/antigen mixtures were incubated at 37 °C for 1 h, together with positive and negative controls. A strongly positive reaction was indicated by the formation of a dark blue ring above a white milk column and any blue layer at the interface of milk and cream should be positive as it might be significant, especially in large herds. The test was considered negative if the color of the underlying milk exceeded that of the cream layer^48,49^.

#### DNA extraction and polymerase chain reaction (PCR)

DNA was extracted from 160 samples (80 milk samples and 80 serum samples) from the same animals (40 bovines and 40 ovines) using the QIAamp DNA Mini kit (QIAGEN, Germany, GmbH) with modifications from the manufacturer’s recommendations. Primers used^16^ were supplied from (BIOBASIC, Canada) and are listed in Table 2. The products of PCR were separated by electrophoresis on 1% agarose gel (APPLICHEM, Germany, GmbH) in 1x Tris/Borate/EDTA (TBE) buffer at room temperature (18–25 °C) using gradients of 5 V/cm. For gel analysis, 15 µl of the products were loaded in each gel slot^50^. A GENERULER 100 bp DNA Ladder (FERMENTAS, THERMO FISHER SCIENTIFIC, Germany) was used to determine the fragment sizes. The gel was photographed by a gel documentation system (ALPHAINNOTECH, BIOMETRA, Germany) and the data was analyzed through computer software.

**Table 2:** The used primer sequences targeting the IS711 gene.

Target gene	Target agent	Primers sequences	Amplified segment (bp)	Reference
IS711	*Brucella* genus	IR1: (5′-GGCGTGTCTGCATTCAACG-3′)	839	^16^
		IR2: (5′-GGCTTGTCTGCATTCAAGG-3′)		

#### Determination of sensitivity, specificity, and accuracy of the newly developed LFIA

The sensitivity, specificity, and accuracy of the newly developed stage have been validated in two subsequent steps:

- 1st : by testing the newly developed LFIA with known Ags standard antisera of *E. coli* O 157, *Y. enterocolitica*, *S. aureus*, *B. abortus*,* B. melitensis*,* B. suis*, and RB51 *B. abortus* vaccine.
- 2nd : the results obtained from the field challenge of the newly developed LFIA were compared to the results obtained from testing the same samples by RBPT, SAT, CFT, I-ELISA, C-ELISA, MRT, and PCR to accurately validate the sensitivity, specificity, and accuracy of the developed LFIA according to^22,31^ and based on the following equations:

$$\:\text{S}\text{e}\text{n}\text{s}\text{i}\text{t}\text{i}\text{v}\text{i}\text{t}\text{y}=\frac{T+}{\left(T+\right)+(F-)}\times\:100\%$$

$$\:\text{S}\text{p}\text{e}\text{c}\text{i}\text{f}\text{i}\text{c}\text{t}\text{y}\:=\frac{T-}{\left(T-\right)+\left(F+\right)}\times\:100\%$$

$$\:\text{A}\text{c}\text{c}\text{u}\text{r}\text{a}\text{c}\text{y}=\frac{\left(T+\right)+\left(T-\right)}{\left(n\right)}\times\:100\%\:$$

$$\:\text{C}\text{o}\text{h}\text{e}\text{n{'}}\text{s}\:\text{K}\text{a}\text{p}\text{p}\text{a}\:\text{c}\text{o}\text{e}\text{f}\text{f}\text{i}\text{c}\text{i}\text{e}\text{n}\text{t}\:\left(K\right)=\frac{Po-Pe}{(1-Pe)\:}$$

where (T+) is the true positive results, (F+) is the false positive results, (F–) is the false negative results, (T–) is the true negative results, (*n*) is the total number, (p_o_) is the relative observed agreement among raters, (p_e_) is the hypothetical probability of chance agreement, and (K) is the Cohen’s Kappa coefficient.

## Results

### Pre-kit-development stage (preliminary stage)

The HPLC analysis of the standard LPS and extracted S-LPS-O revealed distinct chromatographic patterns. The standard exhibited one single peak at a specific retention time. At the same time, the extracted S-LPS-O from *B. abortus* was clarified as a single peak at the same retention time without any impurities. This result indicated a good extraction process with specific chromatographic conditions Supplementary Figs. 1, 2, and 3 and Supplementary Table 1.

The endogenous pyrogen activity of purified S-LPS-O was evaluated using a rabbit pyrogen test. The initial temperatures of the two rabbits were 38.3 °C and 38.4 °C. Injection of S-LPS-O from *B. abortus* S99 raised the temperatures to 39.7 °C and 39.9 °C, respectively. The control rabbit that received PBS as a placebo did not show considerable fluctuation in body temperature.

### Kit-development stage (development and standardization stage)

**Fig. 4:**
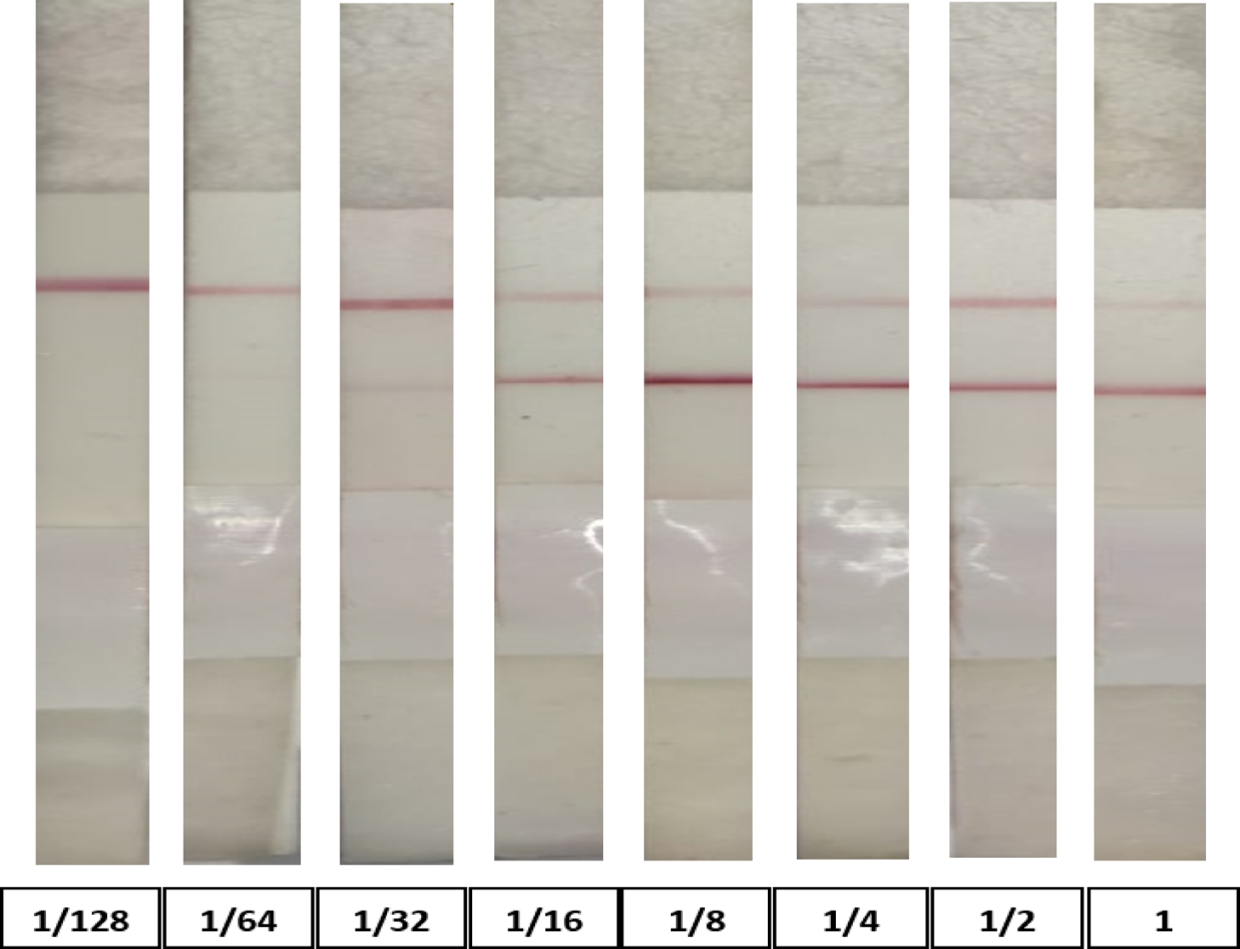
The sensitivity test of LFIA using positive standard antisera of *B. abortus* two-fold serially diluted.

The lower number of antibodies against *Brucella* in the examined serum sample can be detected as a 1.58 S/P ratio ELISA titer/100 µl by using LFIA. RBPT can be detected at a 1.86 sample/positive (S/P) ratio, as shown in Fig. 4; Table 3. The ELISA S/P ratio positive cutoff is 1.20, and the suspect cutoff is 1.1.

**Table 3:** The sensitivity test of LFIA, I-ELISA, C-ELISA, CFT, RBPT, SAT, and MRT using positive standard antisera of *B. melitensis* diluted with 2-fold serial dilution.

Dilution	1	1/2	1/4	1/8	1/16	1/32	1/64	1/128
I-ELISA S/P ratio	2.21 positive	2.04 positive	1.98 positive	1.86 positive	1.58 positive	1.43 positive	1.29 positive	1.15 suspect
C-ELISA	+	+	+	+	+	+	+	+/–
CFT	++	++	++	++	++	+	+	+/–
LFIA	+	+	+	+	+	+	+/–	–
RBPT	+++	+++	++	+	–	–	–	–
SAT	++	++	+	+	–	–	–	–
MRT	+++	+++	++	+	–	–	–	–

### Post-kit development stage (evaluation stage)

**Fig. 5:**
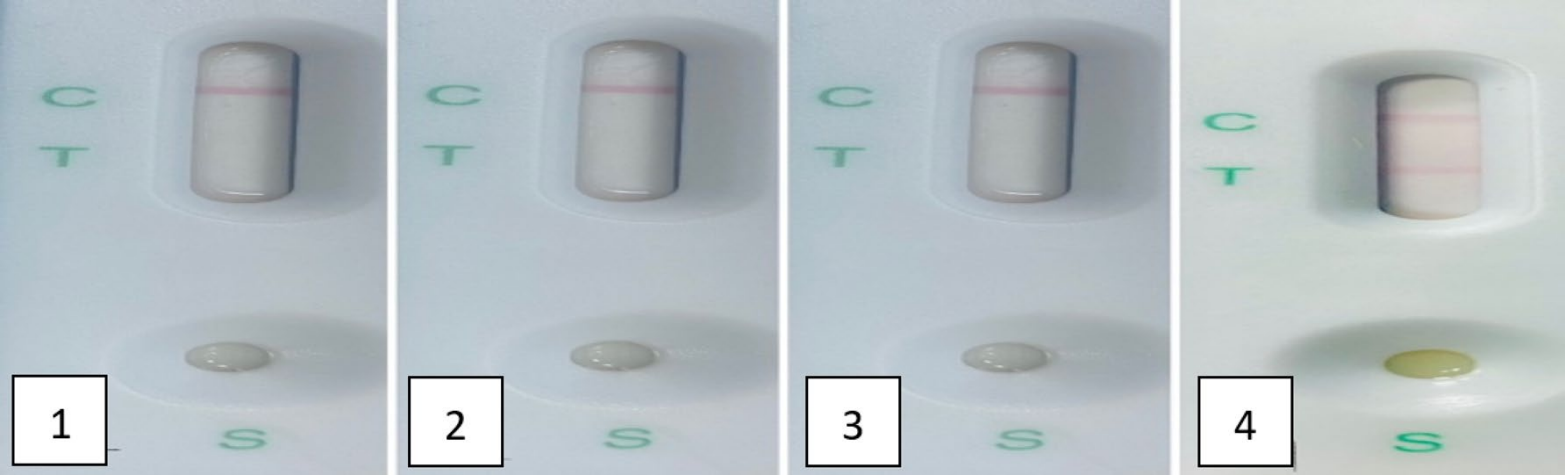
The results obtained through validation of the specificity of the prepared LFIA using standard antisera of *E. coli* O 157, *Y. enterocolitica*, and *S. aureus.*1: positive sera against *E. coli*, 2: positive sera against *Y. enterocolitica*, 3: positive sera against *S. aureus*, and 4: positive sera against *B. abortus*.

**Fig. 6:**
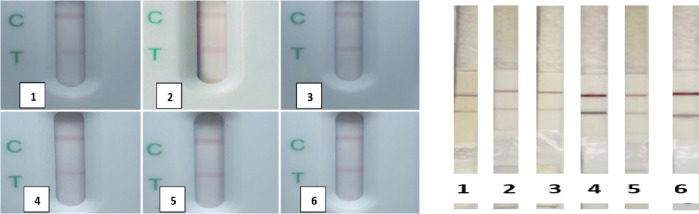
Validation of the sensitivity of the prepared LFIA using antisera of brucellae. 1: bovine serum sample positive *B. abortus* vaccine RB51, 2: bovine serum sample positive *B. melitensis*, 3: bovine serum sample positive *B. abortus*, 4: ovine serum sample positive *B. abortus*, 5: ovine serum sample positive *B. melitensis* and 6: swine serum sample positive *B. suis*.

**Fig. 7:**
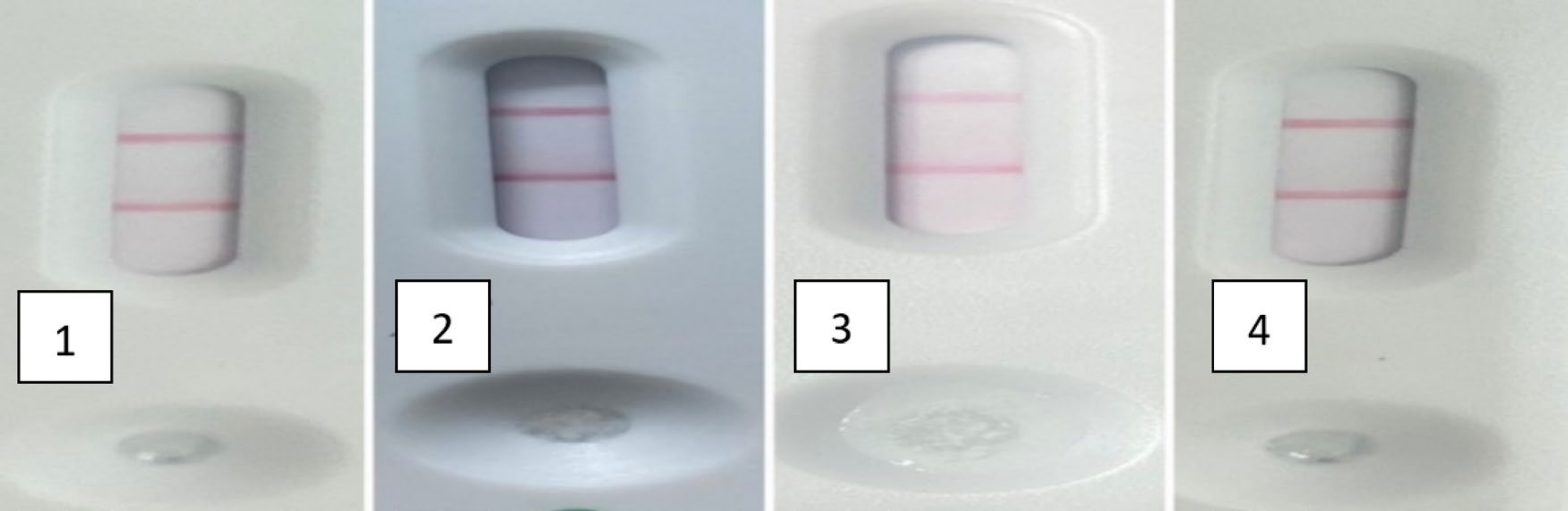
Validation of the sensitivity of the prepared LFIA using milk samples. 1: bovine milk sample positive *B. melitensis*, 2: bovine milk sample positive *B. abortus*, 3, and 4: ovine milk sample positive *B. melitensis.*

The newly developed LFIA was efficiently able to identify the tested standard antisera correctly. The standard antisera of *E. coli* O 157. *Y. enterocolitica*, *S. aureus* gave negative results. But positive *Brucella* spp. antisera gave positive as shown in Fig. 5 and different positive sera/milk from different bovine and ovine vaccinated and infected animals Figs. 6 and 7.

**Fig. 8:**
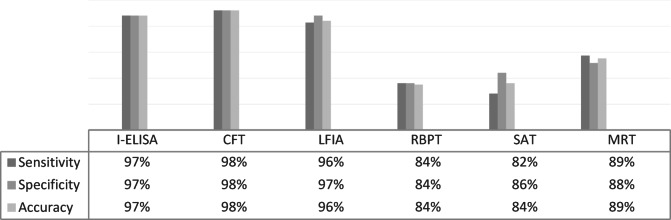
Specificity, sensitivity, and accuracy for the prepared LFIA in comparison with I-ELISA, C-ELISA, CFT, RBPT, SAT, and MRT.

As shown in Fig. 8 the results of LFIA as compared with C-ELISA calculated at T+, F+, F−, and T − were 440, 12, 20, and 398 respectively. So, the sensitivity, specificity, and accuracy of LFIA as compared to C-ELISA were 95.7%, 97%, and 96.3% respectively. The results of I-ELISA as compared with C-ELISA calculated at T+, F+, F−, and T − were 445, 12, 15, and 398 respectively. So, the sensitivity, specificity, and accuracy of I- I-ELISA as compared to C-ELISA were 96.7%, 97%, and 96.9%, respectively. The results of CFT as compared with C-ELISA calculated at T+, F+, F − and T − were 450, 9, 10, and 401 respectively. So, the sensitivity, specificity, and accuracy of CFT as compared to C-ELISA were 97.8%, 97.8%, and 97.8% respectively. The results of RBPT as compared with C-ELISA were 385, 67, 75, and 343 respectively. So, the sensitivity, specificity, and accuracy of RBPT as compared to C-ELISA were 83.7%, 83.7%, and 83.7% respectively. The results of SAT as compared with C-ELISA calculated at T+, F+, F−, and T − were 378, 56, 82, and 354 respectively. So, the sensitivity, specificity, and accuracy of the SAT as compared to C-ELISA were 82.2%, 86.3%, and 84.1% respectively. The results of MRT as compared with LFIA calculated at T+, F+, F−, and T−. So, the sensitivity, specificity, and accuracy of MRT compared to LFIA were 89.3%, 87.87%, and 88.75%.

Results obtained through PCR application on both serum and milk samples that were statistically analyzed through Cohen kappa coefficient calculation was (0.41) which indicates the border value of the “moderate agreement” level between both applications that is ranged in between (0.41–0.60). While PCR on milk samples showed Cohen kappa coefficient calculation as (0.62) which indicates the minimum value of the “substantial agreement” level that is ranged in between (0.61–0.80) with the MRT results. On the other hand, it showed (0.60) Cohen kappa coefficient with the LFIA which indicates the maximum value of the “moderate agreement” level that is ranged in between (0.41–0.60).

## Discussion

Brucellosis is an endemic bacterial zoonosis worldwide and is among the most prioritized zoonotic diseases. It is commonly known that brucellosis has an impact on dairy production and public health in both industrialized and developing nations. The majority of human and livestock cases are not properly detected by the current surveillance systems, which leads to a major underestimation of the disease burden. Additionally, the use of nonspecific antibiotics to treat infected cases may worsen the situation later on, as evidenced by antimicrobial resistance. Due to the clinical, zoonotic, and economic huge effects of brucellosis, several phenotypic, genotypic, and serological diagnostic techniques were developed, and different integral diagnostic protocols were adopted on both national and international control strategies^3,4^. Despite the deep-rooted process of development of brucellosis diagnostics, till now, none of the developed techniques can be considered the sole diagnostic tool for brucellosis that covers all the proper diagnostic criteria at once. Therefore, working in the field of brucellosis diagnosis development and improvement was and remains a very questionable field of science full of gaps that need to be properly filled with a reasonable contribution. The diagnostic approaches for brucellosis can be categorized into three main categories; culture-based phenotypic identification, molecular-based genotypic techniques, and a wide range of conventional serological assays.

Bacterial culture is a gold standard test for confirmation of *Brucella*; however, in addition to a wide range of sensitivity, from 10 to 90%, this method is recognized as a high-risk method for laboratory personnel. Molecular biotechnology techniques such as real-time or Amplification of Multiple Loci by “Abortus, Melitensis, Ovis, and Suis” polymerase chain reaction (AMOS-PCR) are designed to quantitatively detect small amounts of bacterial DNA or differentiate *Brucella* strains which are reliable if precise and expensive equipment is available^51^. In^52^ recorded that the limit of detection for *B. abortus* in most matrices was in the range of 10^3^–10^4^ CFU/g for cultivation and 10^4^–10^5^ CFU/g for direct real-time PCR. Also, the target gene (IS711) confirms the presence of *Brucella* on the genus level: Southern blot analysis determined the distribution of IS711 in the *Brucella* species showed the presence of at least one potentially unique copy of the (IS711) gene in every species except *B. canis*^53^. IR1 and IR2, primer sequences for the *Brucella* genus, were used in the current study on coupled 160 milk and serum samples to correlate the PCR detection of *Brucella* from serum and milk samples with MRT and the newly developed LFIA. The obtained results represent an observable agreement and lower dissociation between the two raters (PCR and MRT) which also can be accounted for the newly developed LFIA due to the low specificity, sensitivity, and agreement of the MRT with the C-ELISA in comparison with the newly developed LFIA.

Intending to develop and improve serological-based diagnostic strategies, research has focused on understanding the pathogenic determinants that allow *Brucella* to establish successful chronic infections and evade immune eradication. Brucellae carry a cell surface lipopolysaccharide whose immunodominant section induces an antibody response that may be difficult to distinguish from that resulting from a true infection, this complicates serodiagnosis because the tests currently used detect antibodies to the LPS^54^. LPS is considered the main bacterial antigen responsible for inducing the expression of pro-inflammatory molecules and the main target for the humoral antibacterial response during the infection of the host^55^. The whole cell or most often the LPS as a major *Brucella* virulence factor, is commonly used for serological assays development; however, cross-reactivity of antibodies against the *Brucella* LPS chain with other Gram-negative bacteria such as *E. coli* O157, *V. cholera*, *F. tularensis*, and *Y. enterocolitica* results in low specificity of these techniques^56–59^.

RBPT, SAT, I-ELISA, and MRT as screening tests and C-ELISA and CFT as confirmatory tests, are the most popular methods that rely on using either whole bacterial antigen or LPS for the detection of anti-*Brucella* antibodies^18^. Also, despite the wide range of testing limitations of MRT, the World Organization of Animal Health (WOAH) recommended MRT for monitoring the occurrence of bovine brucellosis at the herd level, it consists of a screening of specific antibodies against *B. abortus* in bulk milk tanks^60^. The newly developed LFIA showed very competitive comparative results with the MRT by means of sensitivity, specificity, and accuracy with a major advantage which is the different sample types testing applicability in favor of the newly developed LFIA. Also, it has shown better positive and negative predictive values than all compared diagnostic techniques in the current study suggesting that the test is simple, cost-effective, and rapid and provides accurate detection of antibodies to brucellae in the examined samples. This rapid test can therefore be practically implemented in serological screening for brucellosis, although evaluation on a larger scale with various animal sera, blood, and milk samples is still necessary^61^.

Although the currently developed LFIA is not the first lateral flow immunochromatography-based technique to be developed for the diagnosis of brucellosis^22^, competitive advantages-based modifications have been incorporated in the newly introduced LFIA that efficiently reflected on its diagnostic capabilities in comparison with seven different diagnostic assays Table 3; Fig. 8. In the current study, modifications have been employed for the extraction and purification of S-LPS-O from *B. abortus* which would allow early elimination of contaminating components to enhance purity results in highly pure S-LPS-O free of protein and nucleic acids^25^. Using colloidal nanogold which is still the most dominant colorimetric label^62^. As well as using rabbit anti-LPS antibodies on the control line enhance the signal (color intensity) of the control line by additionally binding to the idiotypic determinants (idiotypes) of the anti-idiotypic antibodies of the test serum which consider another novelty point the newly developed LFIA. Recombinant protein A-conjugated nanogold was successfully incorporated, for the first time, as the bioconjugate giving the newly developed LFIA a very important diagnostic advantage which is that the detection ability became not dependent on specific *Brucella* sp., immunoglobulin class, sample type, and/or animal species as protein A has the ability to interact with different immunoglobulins classes of most mammalian species in a wide range of samples. This permits indirect antibody assays with sera for which specific immunoglobulin is not readily available and permits the detection of multiple antigens in indirect immunoassays utilizing antisera from several different species^63^.

## Conclusion

Accurate and rapid diagnosis of re-emerging zoonotic diseases is critical for their control and monitoring. In brucellosis, the early diagnosis of the infected animals is necessary for controlling and eradicating the disease. Therefore, the availability of an ASSURED test that does not require highly sophisticated equipment and laboratories and can be used as a screening field test is of great importance. The developed LFIA offers a powerful and promising point of care (POC) alternative to traditional diagnostic methods for the rapid and accurate diagnosis of brucellosis across different animal species and humans. The study’s findings emphasize the importance of advancing diagnostic technologies for improved disease surveillance and management, particularly in veterinary and clinical settings. Further research is warranted to address persistent challenges in effectively controlling and eradicating brucellosis. This research contributes to the ongoing efforts to develop efficient diagnostic tools for timely disease detection and intervention. The development of robust and efficient diagnostic tools, exemplified by the LFIA with gold nanoparticles (GNPs), offers promising prospects for improved disease management and control strategies for the diagnosis of brucellosis.

## Electronic supplementary material

Below is the link to the electronic supplementary material.

Supplementary Material 1

## Data Availability

All obtained and analyzed data is available through the submitted manuscript. Any further required data is available from the corresponding author upon reasonable request.
