# Supplementary material for: Development and evaluation of a novel lateral flow immunoassay for rapid diagnosis of brucellosis across different animal species
Source: Sci Rep. 2025 Jul 6;15:24149. doi: 10.1038/s41598-025-08741-5 (PMC12230169; doi:10.1038/s41598-025-08741-5)
Supplement: Supplementary file 1 — Supplementary Material 1 [file 41598_2025_8741_MOESM1_ESM.docx]

**
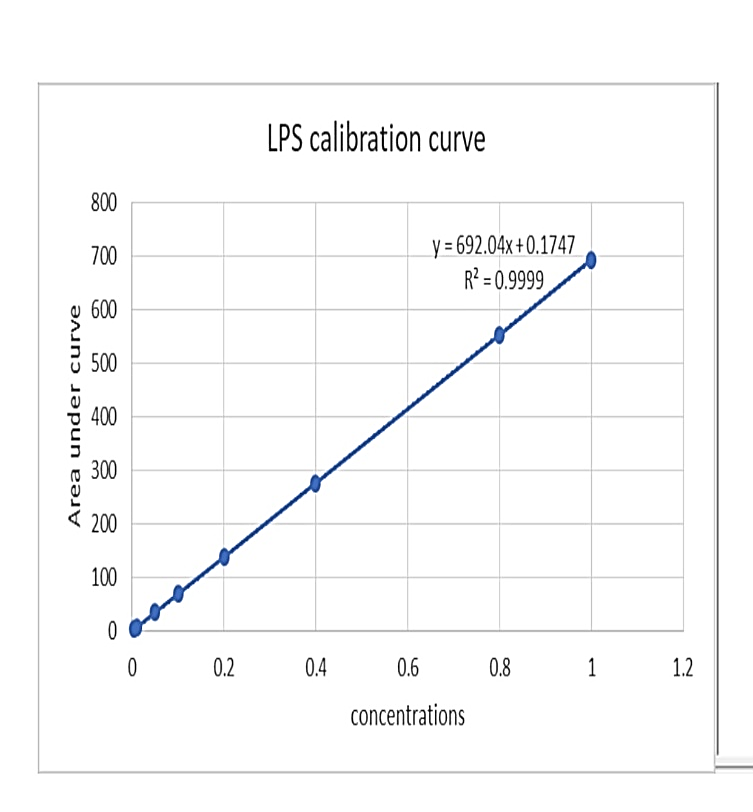
Supplementary Figures and Tables**

**Supplementary Figure (1):** HPLC Chromatogram of LPS standard curve.


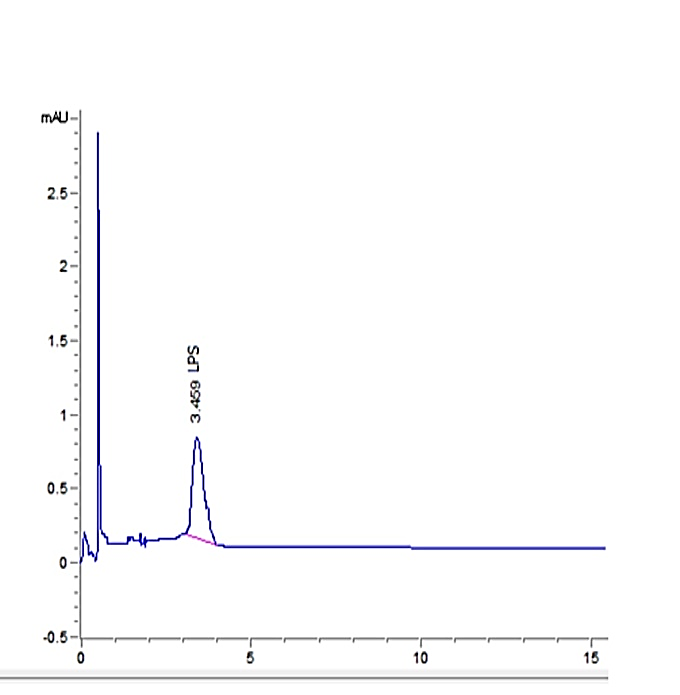


**Supplementary Figure (2):** HPLC Chromatogram of LPS of *Salmonella* Typhimurium concentration standard (0.005 µg/ml).


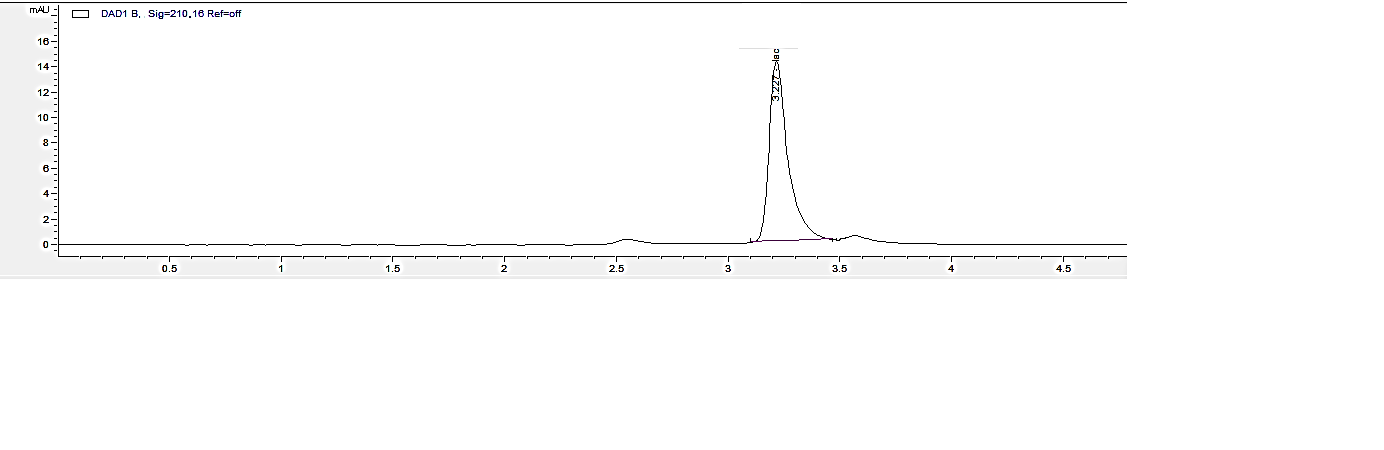


**Supplementary Figure (3):** HPLC chromatogram of the extracted *B. abortus* S-LPS-O with concentration (0.25µg/ml).

**Supplementary Table (1):** calibration table of HPLC chromatogram of the extracted *B. abortus* S-LPS-O.

| # | RT | Signal | Compound | Lvl | Amt [ng/ μl] | Area | Rsp.Factor | Ref | ISTD | # |
| --- | --- | --- | --- | --- | --- | --- | --- | --- | --- | --- |
| 1 | 3.223 | DAD1 B | lac | 1 | 2.000 | 11.887 | 1.6825e-1 | No | No |  |
|  |  |  |  | 2 | 5.000 | 30.367 | 1.6465e-1 |  |  |  |
|  |  |  |  | 3 | 10.000 | 59.915 | 1.6690e-1 |  |  |  |
|  |  |  |  | 4 | 20.000 | 119.180 | 1.6782e-1 |  |  |  |
|  |  |  |  | 5 | 50.000 | 308.980 | 1.6182e-1 |  |  |  |
